# Slender women and overweight men: gender differences in the educational gradient in body weight in South Korea

**DOI:** 10.1186/s12939-017-0685-9

**Published:** 2017-11-21

**Authors:** Yeonjin Lee

**Affiliations:** National University of Singapore, 10 Kent Ridge Crescent, Singapore, 119260 Singapore

**Keywords:** Body weight, Weight perception, Education, Gender, Health behaviors

## Abstract

**Background:**

Little is known about the gender-specific mechanisms through which education is associated with weight status in societies that have experienced a rapid rise in their obesity rates. This study extends previous literature by examining how the link between education and weight status operates within the structure of gender relations in South Korea where huge gender differences have been observed in the educational inequalities in weight status.

**Methods:**

Using the Korean National Health Survey (*N* = 17,947) conducted in 2008–2012 conditional quantile regression models were estimated to assess the associations between education and body weight distribution. The mean difference in the predicted probabilities of perceiving body image as average was compared by educational attainment for women and men while setting all other covariates at their means.

**Results:**

Highly educated women were more likely to utilize their human capital to obtain slender body shape and the relationship was not mediated by economic resources. In contrast, education was positively associated with being overweight and obesity among men, for whom behaviors promoting healthy weight often conflict with a collective ideology at work that strongly supports long work hours and heavy alcohol consumption. Furthermore, Korean men were more likely to under-perceive their body size than Korean women, that is, overweight men tend to consider themselves to be of ‘average’ weight, regardless of their educational attainment.

**Conclusions:**

Current study found that gender inequalities in social status in South Korea operate to affect the relationship between education and weight status among men and women in unique ways. Weight status can be socially patterned by the interplay between education, economic, and behavioral resources within the structure of gender relations.

## Background

Obesity has become increasingly prevalent in countries where it was rarely a problem in the past. Several factors account for this, including these countries experiencing remarkable economic growth, and the emergence of an obesogenic environment [1, 2]. The emergence of obesogenic environments makes the human agency and knowledge that accrue from schooling more important for obesity prevention as people are exposed to fast changes in physical activity patterns and dietary intake [3].

Obesity is typically more prevalent among women because women’s bodies are adapted to store more fat due to biological factors related to reproduction [4]. The experience of childbirth also significantly increases the probability of obesity [5]. Recent studies have documented that the gender differences in being overweight and obesity prevalence vary by social and cultural contexts, which indicates that gender is an important social factor of obesity as well as a biological marker [6, 7].

Research from Western countries links higher education with a lower risk of obesity but whether the shape and strength of the relationship varies by gender is inconclusive [8]. Relatively less is known about the gender-specific mechanisms through which education is associated with weight status in non-Western societies that have experienced a rapid rise in their obesity rates. Men and women are exposed to higher education in different ways and gain unequal socioeconomic returns to their levels of education in the societies with a rigid gender hierarchy [9, 10]. Furthermore, there are potential gender differences in social consequences of obesity [11], and in the cultural ideologies regarding body shape [12], which contribute to generating different motivation for men and women to manage the emerged obesogenic environments.

Higher levels of education enable people to be more efficient in health production, for example, more educated people may be better able to practice an efficient allocation of caloric intake and expenditure [13, 14]. The effect of education is indirectly attributable to economic resources that may protect them against risk factors for obesity. Education is also closely associated with cultural capital and predisposes people to be knowledgeable about normative codes of desirable physical appearance [15]. Therefore, the variation in the educational gradients in weight status among men and women may be reflective of gender-linked differences in normative cultural codes regarding ideal body size. Social pressure to conform to ideal body shape may vary between men and women [16]. In developed societies, obese individuals are often stigmatized and social penalties for being obese are greater for women than for men [17]. The culture that conveys ubiquitous images of female celebrities with thin bodies also encourages people to consider slimness as part of feminine beauty and as a characteristic of social distinction [18, 19]. Women are more sensitive to their body shape particularly in anti-egalitarian countries where they are more likely to be evaluated by societal conventions based on physical appearance than men. In these societies, women’s beauty is more likely to be valued as a symbolic asset, e.g., a beauty premium, which translates into tangible social resources in the labor and marriage market [20, 21].

South Korea (hereafter ‘Korea’) has experienced a rapid economic development. State-led industrialization has led to an enormous occupational transition from rural primary product sectors to service and manufacturing sectors, which have contributed to reduced physical activity [22]. Access to high-calorie foods became easier and cheaper through the growth of convenience food stores and restaurants [23]. Due to fast social change, most Korean adults who spent early childhood without knowledge of obesity are now facing an obesity epidemic.

Even if the prevalence of obesity in Korea is relatively moderate compared to other high- and middle-income countries (<30% according to Yoon et al., 2006), it has been growing quickly, especially among middle-aged men [1]. The statistics from the Korean National Health Survey showed that the prevalence of people whose Body Mass Index (BMI) is over 25 kg/m^2^ significantly increased over the last two decades from 13.9% in 1995 to 31.5% in 2014. Although much slower increase in obesity has been observed among women in Korea [24] severe educational disparities in weight status have been observed for women in recent years [25]. According to the cross-national statistics among the 11 Organisation for Economic Co-operation and Development (OECD) countries, the largest educational differences in being overweight and obesity have been observed for Korean women along with Spanish women, while the smallest educational inequalities have been found for Korean men [26]. Despite the stark gender differences in educational gradients in weight status, little is known about what creates the variations between men and women in this society.

Despite the expansion of higher education, which has reduced the gender gap in educational attainment, gender inequalities in labor force participation, wages, and type of employment remain substantial in Korea. According to the “glass-ceiling index” of the Economist (2016) based on the latest cross-national data, Korea is the worst place to be a working woman among 30 OECD countries. Korea has the largest gender wage gap and Korean women are least likely to be in senior managerial positions, which is far behind women in Scandinavian countries [27]. Most Korean women do not obtain the same level of economic rewards and autonomy as men of a similar level of education. The report issued by the Korea Statistical Information Service (KOSIS) in 2016 showed that only about 53% of working-aged Korean women (aged 15 and older) participate in the labor force, compared to 74.7% of men [28]. Thus, it is possible that for women the effect of education may not be attributable to economic resources and play a direct role in determining health and health behaviors. Furthermore, studies have documented significant barriers in the Korean labor market that contribute to the interruption of occupational careers of women upon marriage and childbearing [9]. In the context of a strong gender division of labor, men’s earnings may be a particularly crucial factor in marriage markets and a married woman’s social status is likely to be determined by her husband’s social status [29]. Therefore, women’s bodies are often sexually objectified by men in the process of mate selection, which may drive women toward investing in human capital to achieve physical attractiveness [30, 31]. On the other hand, better educated Korean men are less likely to be under appearance-related social pressures and less likely to be sensitive to body size [32].

Health-related lifestyles are sometimes at odds with the ideology of the groups or organizations that people belong to [33]. Individuals are expected to conform to the group’s dominant ideology in order to avoid any possible disadvantages from not fulfilling the normative rules [34]. Collective practices in Korea support the norm favoring longer working hours. The 2014 OECD statistics show that Korea has the longest working hours compared to other high income countries [35]. Long working hours decrease workers’ time for outdoor activities and sleep which play a significant role in the etiology of obesity [36]. Korean male workers are also under pressure to participate in drinking gatherings as an extension of formal working hours to improve work place relations [37]. Embedded in a cultural system of social interactions, alcohol consumption, a ‘social lubricant’, is positively perceived by professional and sales workers as a way to show solidarity with one’s business network and to ease the stress associated with work [38]. Heavy drinking falls into a line of masculine icons in Asian culture while it places a taboo on women who drink [39]. Unhealthy behaviors such as sitting behind a desk all day, overworking while reducing leisure time, and binge drinking have been supported as a virtue shared by elite groups that measure social success among Korean men of working age. This may lead Korean men with college educations, who are generally more likely to have sedentary jobs and higher incomes than men with low levels of education, to be exposed to higher behavioral risks for gaining weight [40]. Thus, education may not be a strong predictor of being overweight and obesity among Korean men after controlling for economic resources, for whom behaviors promoting healthy weight often conflict with a collective ideology at work [38], and for whom motivation to obtain professional success is stronger than a desire for better appearance.

Using repetitive cross-sectional data from 2008 to 2012, this study extends previous literature by examining how the link between education and body weight distribution operates before and after controlling for other socioeconomic characteristics and health behaviors within the structure of gender relations in Korea. We also hypothesize that Korean men are more likely to under-estimate their body weight compared to Korean women and the educational gradient in weight perception will be less pronounced among men than women. We investigate whether Korean women with higher educational attainment are more likely to perceive their body weight as being higher than their actual weight status and women with lower educational attainment perceive their weight as being lower than their actual weight status.

## Methods

### Data and measures

The data for this study come from the Korean National Health and Nutrition Examination Survey (KNHANES). The KNHANES is a nationally representative survey, consisting of a combination of a health interview, a nutritional survey, and health examinations with comprehensive information on biochemical and anthropometric measurements, health status, health behaviors and socio-demographic characteristics. Standardized health examinations were carried out in a specially equipped mobile examination centers. KNHANES is a cross-sectional survey of Korean individuals chosen in a multistage clustered sampling frame based on geographical unit, sex and age, using the 2005 National Census Registry. Since the KNHANES examines independent sets of randomly selected individuals, it very rarely happens to select the same person repeatedly in the following year.

The current research utilized five waves of KNHANES from 2008 to 2012. The sample was restricted to individuals ages 25–64. By age 25 most people have completed their highest level of education, and most individuals ages 25–64, especially men, participated in the labor force at the time of interview. From the pooled weighted KNHANES sample of 19,978, women who were pregnant at the time of the examination were excluded (*N* = 153). Individuals who had missing information on measured weight (*N* = 68) were deleted. Given a large enough sample, around 9% of the cases with missing information on the explanatory variables were removed without imputation [41]. The study found that there were no significant associations between the variables with missing data and other key variables. Deleting cases also did not bias result displayed in this paper. The final sample size consists of 17,947 individuals of whom 7109 were men and 10,838 were women.

### Dependent variables

The current study used Body Mass Index (BMI = weight in kg/ (height in meters)^2^) as an indicator of weight status. Height and weight were measured in light indoor clothing without shoes to the nearest 0.1 kg and 0.1 cm respectively. BMI can be categorized based on the criteria recommended by the World Health Organization (WHO) for the study of obesity among Asians as follows: underweight (BMI less than 18.5 kg/m^2^), normal weight (18.5 ≤ BMI < 23 kg/m^2^), overweight (23 ≤ BMI < 27.5 kg/m^2^), and obesity (BMI higher than 27.5 kg/m^2^) [42]. The appropriateness of using standard BMI cutoff points of overweight and obesity has been controversial given the fact that adiposity tissue volume and lifestyle factors are heterogeneous across populations [43]. Given that there is no universal agreement about what BMI cutoff points should be used to define overweight or obesity for a specific population, this study considers BMI as a continuous variable and categorized BMI is used as an outcome variable of sensitivity analyses.

Weight perception was measured based on a question asking how individuals perceived their own body size with a five-point scale ranging from ‘underweight’ to ‘overweight’ (1: underweight, 2: lighter than average, 3: about average, 4: slightly overweight, 5: overweight). This measure of weight perception was coded into three categories to obtain an adequate number of cases in each category (1: perceived lighter than average (perceived underweight) (1 + 2), 2: perceived average (3), 3: perceived overweight (4 + 5)).

### Explanatory variables

The study adjusted for the following demographic characteristics: age, year of survey, marital status (currently married, separated/divorced/widowed, never married), self-rated health (poor, fair, good), as well as region of residence (metropolitan cities, small cities, rural areas). The key explanatory variable is educational attainment coded as follows: middle school or less, high school, and at least some college level education. We included annual household income and current occupational status as economic resources. Annual household income was derived from the income of all household members earned during the last year and was treated as a continuous variable. Household income was logged because it was skewed and it had a higher R-square compared to unlogged household income. The household income was based on Korean currency; 1 dollar = 1020 won. The respondent’s occupation was coded into four categories based on industry: professional work; clerical/service/sales work; manual work; and unemployed/housewives/students. Manual work included work in agriculture, forestry, fisheries, and other manual labor. To test whether health behaviors play an important role in the link between education, economic resources, and body weight, we included hours of working, smoking behavior, and alcohol consumption. Average hours of working was self-reported and coded as a continuous variable ranging from 0 to 10 or more hours. Working hour was reported based on average duration of working per day during the last 12 months. Respondents were categorized into one of three smoking groups: never smoked, former smoker, and current smoker. Measures of current drinking patterns were based on self-reports on frequency of drinking and amount of alcohol consumed per day: (1) never drink, (2) drink less than 30 g ethanol per day on average and once a month, (3) drink less than 30 g ethanol per day on average at least twice a month, (4) drink about 40–60 g ethanol per day on average, (5) drink about 60–80 g ethanol per day on average, (6) drink more than 80 g ethanol per day on average.

The correlation between education and weight could be generated by common hereditary or health endowment that affects both schooling and obesity [44]. Therefore, we additionally controlled for parental education as a measure of parental socioeconomic status to account for family backgrounds. Parental educational attainment was coded based on the highest level of school completed. Because of the high correlation between the father’s and the mother’s educational attainment, we generated a categorical indicator of parental education by using the highest educational attainment of either the father or the mother: no school, lower secondary, and upper secondary or above.

## Statistical analysis

To examine gender differences in the distribution of the explanatory variables, t-tests were used for quantitative variables and chi-square tests were used for categorical variables. Conditional quantile regression model was adopted to estimate the effect of education on the entire distribution of BMI, not merely on its conditional mean. The quantile regression method focuses on the educational gradients in both the right and the left tail of the BMI distributions known to be related with high health risks [45]. For instance, the education gradient may be steeper at the right tail of the BMI distributions, which indicates that higher education offers greater protection against weight gain in overweight groups. This approach also properly deals with nonlinearities in the relationships of BMI with its predictors. We assessed whether the coefficients of education vary at high versus low quantiles of body weight and how they change before and after controlling for potential mediators. Current study adopted a bootstrap method to estimate standard errors with 100 replications. All models were estimated separately for men and women. Model 1 evaluates the bivariate relationship between educational attainment and BMI. Model 2 includes demographic characteristics (e.g., age, year of survey, marital status, and region of residence) and income and occupation as plausible economic mediators. In model 3, health behaviors are added to model 2 to examine whether behavioral mediators further explain the associations between education, economic and occupational status, and BMI. Model 2 and 3 also control for parental education and self-rated health, which may affect respondents’ educational attainment and health behaviors, and reflect family backgrounds. We performed sensitivity analyses that compared alternative way of coding the BMI variable. Using multinomial logit regressions, we assessed the associations between BMI categories, educational attainment, and the hypothesized pathway variables. The models compared underweight, overweight, and obese categories relative to the reference category of normal weight.

Second, to see whether there are educational gradients in weight perception among men and women, the analysis regressed weight perception on age, year of survey, education, BMI categories (underweight, normal, overweight, obesity), and interaction terms between education and the BMI categories. The estimates were weighted by survey weights to be nationally representative and account for the complex sampling design of KNHANES. The study produced predicted probabilities of whether people perceive themselves as having “average” weight using the results of the multinomial logit regressions. The mean difference in the predicted probabilities of perceiving body image as average was compared by educational attainment and BMI categories for women and men while setting all other covariates at their means. All models were estimated in Stata 12.

## Results

### Sample characteristics

Table 1 provides sample characteristics including weighted means for continuous variables, and percentages for categorical variables for men and women. With respect to weight status, men were more likely to be overweight than women whereas women were more likely to be underweight than men. For example, 10.9% of women and 14.0% of men were obese; 36.9% of women and 51.2% of men were overweight; the percentages of underweight were 5.7% for women and 2.4% for men (Table 1). With respect to weight perception, around 50.5% of women perceived that they were heavier than average compared to about 42.6% of the men despite the fact that men were more likely to be overweight or obese than women.

**Table 1:** Sample characteristics by gender, ages 25–64, distribution (%) or mean (SD), 2008–2012 Korean National Health and Nutrition Examination Survey (KNHANES), (*N* = 17,947)

Characteristics	Total (N = 17,947)	Women (*N* = 10,838)	Men (*N* = 7109)	*p*-value*
Weight status				
Underweight (BMI < 18.5)	4.0	5.7	2.4	0.00
Normal weight (18.5 ≤ BMI < 23)	39.3	46.6	32.3	
Overweight (23 ≤ BMI < 27.5)	44.2	36.9	51.3	
Obese (27.5 ≤ BMI)	12.5	10.9	14.0	
BMI (continuous)	23.8 (3.4)	23.2 (3.5)	24.3 (3.2)	0.00
Self-perceived weight status				
Lighter than average	15.1	10.2	19.9	0.00
Average	38.4	39.4	37.6	
Heavier than average	46.5	50.5	42.6	
Self-rated health				
Poor	16.0	18.8	13.3	0.00
Fair	45.5	45.9	45.2	
Good	38.4	35.3	41.4	
Socio-demographic characteristics				
Educational attainment				
Middle school	21.0	25.3	16.9	0.00
High school	39.8	40.6	39.0	
Some college and above	39.2	34.2	44.0	
Age	42.9 (10.6)	43.1 (10.6)	42.6 (10.6)	0.00
Parent’s education				
No school	14.5	15.0	14.1	0.16
Lower secondary	51.2	51.7	50.7	
Upper secondary/above	34.3	33.4	35.2	
Marital status				
Single	15.8	11.2	20.3	0.00
Married	77.8	80.0	75.7	
Separated/divorced/widowed	6.4	4.0	8.8	
Region of residence				
Metropolitan cities	47.9	49.0	46.9	0.01
Small cities	34.6	34.8	34.5	
Rural area	17.4	16.2	18.6	
Annual household income (10,000 won)	5222.4 (10,197.0)	5193.5 (10,651.0)	5250.2 (9737.6)	0.78
Occupational status				
Professional worker	14.4	12.5	21.7	0.00
Clerical/service/sales worker	25.9	25.0	27.0	
Manual worker	27.8	16.8	38.0	
Not in the labor force	31.9	45.7	13.3	
Health behaviors				
Hours of work	6.2 (3.5)	5.1 (3.4)	8.0 (2.9)	0.00
Smoking				
Current Smoker	35.2	8.4	61.1	0.00
Former smoker	12.5	4.0	20.6	
Non-smoker	52.4	87.6	18.3	
Drinking				
Never	20.3	29.3	11.7	0.00
Light drinker	24.7	36.3	13.6	
Light and frequent drinker	16.7	19.0	14.5	
Moderate drinker	14.4	8.8	19.7	
Heavy drinker (60–80 g ethanol/a day)	11.6	3.7	19.2	
Binge drinker (> 80 g ethanol/a day)	12.2	2.9	21.3	

*p-value refers to the difference in the distribution of the explanatory variable between women and men based on a chi-square test for categorical variables and t-test for continuous variables

Women were socioeconomically disadvantaged compared to men. Men were more highly educated than women; 44.0% of men had attended at least some college while 34.2% of women had done so. Higher percentage of women (25.3%) than men (16.9%) had only less than middle school education. Men were significantly more likely to be working in professional jobs than women (21.7% vs. 12.6%). In contrast, almost half of the women (45.7%) were out of the labor force. Gender difference in household income was not significant since household income included all possible sources of income from family members and there were only a small number of women head of household. Thus, income of women who were not currently in the labor force may reflect their husbands’ income.

The distribution of health behaviors significantly differed by gender. Men reported significantly longer mean work durations than women. On average women reported work duration of 5.1 h and men reported 8 h of work. Among women 8.4% reported that they were current smokers, whereas 61.1% of men reported that they were currently smoking. Around 2.9% of women reported that they were heavy drinkers drinking more than 80 g ethanol/a day compared to 21.3% of the men. In contrast, 29.3% of women never drank; the respective percentage for men was only 11.7%.

Table 2 presents results from the conditional quantile regression models examining whether the effect of educational attainment differed along the 10th, 25th, 50th, 75th, and 90th percentiles of the BMI distribution for women. Educational attainment was a significant predictor of body weight among women such that women with higher levels of schooling were significantly less likely to gain weight. The negative college level education coefficients were larger at the higher quantiles of BMI, which suggests that college education had a larger protective effect at the higher quantiles of BMI (i.e. women who are overweight) and there were larger educational inequalities at the right tail of the BMI distribution. For example, in Model 1 without confounders and mediators, the 10th percentile of BMI for women with college level education was −1.07 BMI units lower than for women with middle school level educations and at the 90th percentile, the coefficient for the BMI of women who had a college degree was −2.42. The negative education gradient persisted even after controlling for annual household income and occupational status (Model 2). For women in the 10th percentile, the difference in BMI between those with some college level education and those with middle school or lower level of schooling was 65%, while for those in the 90th percentile it was 86%. Women in the higher BMI percentiles with higher household income were significantly less likely to be obese but occupational status was not a significant predictor of body weight among them. Adding controls for weight-related behaviors, including mean daily working hours, smoking, and alcohol consumption, in Model 3 barely explained the protective effect of higher education against obesity among women. Working hour was a significant predictor of body weight for women in the higher BMI percentiles while drinking habit was not a significant predictor of obesity. Based on the above results, educational attainment appears to be the most important social determinant of weight status among Korean women. An interaction term with age group and educational attainment was found to be insignificant in a fully adjusted model and did not improve the model fit, which indicates that the association between education and weight status did not vary by age (Wald test*: p =* 0.149; Likelihood ratio test: *p = 0.102*).

**Table 2:** Coefficients from conditional quantile regression models on body mass index (BMI) among women (N = 10,838), 2008–2012

Characteristic	10th Quantile	25th Quantile	50th Quantile	75th Quantile	90th Quantile
Model 1:					
Educational attainment (middle school or less)^a^					
High school	−0.52(0.11)**	−0.60(0.11)**	−0.80(0.14)**	−0.75(0.14)**	−0.84(0.17)**
College and above	−1.07(0.14)**	−1.25(0.12)**	−1.56(0.15)**	−2.05(0.19)**	−2.42(0.20)**
Goodness of fit: R1(τ)	0.08	0.10	0.07	0.05	0.03
Model 2:					
Educational attainment (middle school or less)^a^					
High school	−0.57(0.11)**	−0.65(0.10)**	−0.74(0.12)**	−0.62(0.15)**	−0.58(0.20)**
College and above	−1.04(0.14)**	−1.25(0.15)**	−1.39(0.10)**	−1.70(0.14)**	−1.99(0.22)**
Household income (logged)	−0.03(0.04)	0.05(0.04)	−0.04(0.03)	−0.27(0.06)**	−0.48(0.08)**
Occupational status (manual worker)					
Professional	0.09(0.18)	0.17(0.16)	−0.05(0.14)	−0.08(0.20)	0.14(0.29)
Clerical/service/sales	0.32(0.12)*	0.27(0.12)*	0.02(0.11)	0.16(0.12)	0.26(0.21)
Not in the labor force	0.16(0.09)	0.14(0.08)	−0.11(0.10)	−0.03(0.10)	0.15(0.17)
Goodness of fit: R1(τ)	0.09	0.10	0.07	0.06	0.04
Model 3:					
Educational attainment (middle school or less)^a^					
High school	−0.59(0.10)**	−0.61(0.09)**	−0.71(0.11)**	−0.57(0.13)**	−0.49(0.21)*
College and above	−1.04(0.11)**	−1.20(0.11)**	−1.32(0.12)**	−1.63(0.16)**	−1.81(0.21)**
Household income (logged)	−0.04(0.05)	0.01(0.03)	−0.04 (0.03)	−0.26(0.06)**	−0.46(0.05)**
Occupational status (manual worker)					
Professional	0.14(0.19)	0.12(0.15)	−0.08(0.15)	−0.05(0.20)	0.04(0.35)
Clerical/service/sales	0.35(0.13)**	0.18(0.11)	0.05(0.12)	0.12(0.12)	0.17(0.20)
Not in the labor force	0.39(0.22)	0.29(0.14)*	0.02(0.13)	0.24(0.21)	0.48(0.22)*
Health behaviors					
Hours of work	0.03(0.02)	0.03 (0.03)	0.02(0.02)	0.05(0.02)*	0.05 (0.02)*
Smoking (non-smoker)					
Current smoker	−0.40(0.11)**	−0.33(0.15)*	−0.23(0.15)	−0.03(0.23)	0.29(0.48)
Former smoker	0.12(0.10)	0.32(0.18)	0.44(0.18)*	0.31(0.28)	0.20(0.32)
Drinking (never)					
Light drinker	0.19(0.08)*	0.22(0.08)**	0.05(0.10)	−0.10(0.12)	0.00(0.15)
Light and frequent drinker	0.33(0.08)**	0.37(0.08)**	0.28(0.12)*	0.05(0.13)	0.27(0.23)
Moderate drinker	0.48(0.13)**	0.52(0.13)**	0.31(0.17)	0.27(0.25)	0.25(0.36)
Heavy drinker (60–80 g ethanol/a day)	0.53(0.24)*	0.40(0.20)	0.47(0.30)	0.43(0.30)	0.59(0.56)
Binge drinker (> 80 g ethanol/a day)	0.61(0.22)**	0.92(0.31)**	1.15(0.20)**	1.40(0.44)**	2.04(0.70)**
Goodness of fit: R1(τ)	0.09	0.10	0.08	0.06	0.04

*p*-value ** < 0.01; * < 0.05. Age, survey year, marital status, self-rated health, region of residence, and parental education are controlled for in the model 2 and 3. Bootstrap standard errors are in parentheses

^a^Omitted category is shown in parenthesis

Table 3 displays the results for men. As seen in Model 1, educational attainment among men was positively associated with the risk of gaining weight across the entire BMI distribution. Model 2 in Table 3 shows that household income and occupational status substantially attenuated the magnitudes of the coefficients of education, and education was no longer statistically significantly associated with body weight. At the same time, more economically advantaged men in lower and mean BMI percentile were more likely to gain weight. As hypothesized, men who work in the clerical/service/sales and professional occupations were significantly more likely to gain weight compared to men with manual jobs and this tendency was not substantially heterogeneous according to the BMI distribution. In separate models that compare the coefficients of schooling after controlling for household income and occupational status respectively, the study found that of the two socioeconomic characteristics, occupation played a more crucial role in explaining the association between educational attainment and being overweight and obesity among men. When health behaviors were additionally adjusted for in Model 3, the relationship between professional jobs and clerical/service/sales jobs and BMI became non-significant for men in the 90th percentile (BMI = 28.1). Additional analyses showed that men with clerical/service/sales jobs were more likely to engage in chronic, heavy drinking and professional workers were likely to have longer working hours relative to manual workers. The formal test of mediation also showed that obese men with professional and clerk/service-related jobs were more likely to gain weight because of their binge drinking habits and longer working hours (results not shown). Behavioral pathway did not fully explain the relationship between occupation and weight status of men in the lower BMI percentiles; unexplained pathways may include work-related psychological stress, calorie intakes, and lack of physical activity. An interaction term with age and education was not statistically significant in a fully adjusted model among men and did not improve model fit as was the case with women (Wald test: *p* = 0.239; Likelihood ratio test: *p* = 0.671).

**Table 3:** Coefficients from conditional quantile regression models on body mass index (BMI) among men (N = 7109), 2008–2012

Characteristic	10th Quantile	25th Quantile	50th Quantile	75th Quantile	90th Quantile
Model 1:					
Educational attainment (middle school or less)^a^					
High school	0.15(0.18)	0.04(0.10)	0.19(0.09)*	0.16(0.12)	0.42(0.20)*
College and above	0.46(0.19)*	0.32(0.11)**	0.36(0.12)**	0.46(0.11)**	0.59(0.21)**
Goodness of fit: R1(τ)	0.01	0.01	0.01	0.01	0.01
Model 2:					
Educational attainment (middle school or less)^a^					
High school	0.06(0.15)	−0.02(0.16)	0.01(0.15)	−0.03(0.16)	−0.01 (0.21)
College and above	0.13(0.16)	−0.04(0.16)	0.02(0.15)	−0.03(0.24)	−0.10(0.17)
Household income (logged)	0.39(0.10)**	0.30(0.05)**	0.19(0.09)*	0.06(0.05)	0.11(0.11)
Occupational status (manual worker)					
Professional	0.46(0.15)**	0.59(0.16)**	0.52(0.11)**	0.39(0.12)**	0.44(0.21)*
Clerical/service/sales	0.33(0.11)**	0.47(0.12)**	0.46(0.10)**	0.40(0.12)**	0.50(0.19)**
Not in the labor force	−0.04(0.22)	0.18(0.18)	0.17(0.18)	−0.16(0.16)	−0.07(0.26)
Goodness of fit: R1(τ)	0.02	0.01	0.01	0.01	0.02
Model 3:					
Educational attainment (middle school or less)^a^					
High school	−0.04(0.22)	0.03(0.12)	0.08(0.14)	−0.03 (0.15)	−0.09(0.17)
College and above	−0.01(0.32)	0.09(0.16)	0.07(0.17)	0.05(0.19)	−0.02(0.24)
Household income (logged)	0.39(0.10)**	0.31(0.05)**	0.10(0.06)	0.03(0.07)	0.08(0.12)
Occupational status (manual worker)					
Professional	0.32(0.23)	0.42(0.16)**	0.50(0.12)**	0.41(0.14)**	0.36(0.21)
Clerical/service/sales	0.36(0.15)*	0.33(0.09)**	0.36 (0.12)**	0.30(0.11)**	0.38(0.23)
Not in the labor force	−0.16(0.28)	0.01(0.21)	0.17(0.22)	0.16(0.21)	0.25(0.32)
Health behaviors					
Hours of work	−0.02(0.03)	−0.02 (0.02)	0.02(0.03)	0.01(0.01)	0.08(0.03)*
Smoking (current smoker)					
Non-smoker	0.43(0.16)**	0.24(0.13)	0.20(0.15)	0.17(0.15)	0.22(0.17)
Former smoker	0.64(0.18)**	0.63(0.13)**	0.57(0.11)**	−0.02(0.14)	0.11(0.19)
Drinking (never)					
Light drinker	0.10(0.20)	0.31(0.19)	0.47(0.14)**	0.44(0.23)	0.63(0.45)
Light and frequent drinker	0.20(0.15)	0.13(0.15)	0.25(0.21)	0.02(0.18)	0.22(0.31)
Moderate drinker	0.31(0.14)*	0.39(0.17)*	0.54(0.15)**	0.26(0.18)	0.12(0.20)
Heavy drinker (60–80 g ethanol/a day)	0.62(0.16)*	0.67(0.19)**	0.75(0.14)**	0.63(0.16)**	0.69(0.24)**
Binge drinker (> 80 g ethanol/a day)	1.11(0.17)**	1.26(0.21)**	1.32(0.18)**	1.17(0.19)**	1.37(0.25)**
Goodness of fit: R1(τ)	0.04	0.03	0.02	0.02	0.03

*p*-value ** < 0.01; * < 0.05. Age, survey year, marital status, self-rated health, region of residence, and parental education are controlled for in the model 2 and 3. Bootstrap standard errors are in parentheses

^a^Omitted category is shown in parenthesis

The results of multinomial regressions demonstrated that the associations between educational attainment and overweight and obesity were not sensitive to the way BMI was categorized for both women and men. Educational attainment was a significant predictor of weight status among women such that women with higher levels of schooling were significantly less likely to be overweight or obese. Korean men who have higher educational attainment are more likely to be overweight and obese than men who have lower educational attainment and the relationship between educational attainment and body weight status will be at least partly explained by occupation. The detailed results of the sensitivity analyses are presented in Appendix. We additionally tested whether the shape of the educational gradient in weight is uniquely patterned for women in their 20s and 30s given that this group is likely to be more sensitive to body size. However, the strength and shape of the link between education and weight status for women in their 20s and 30s were not different from those of older counterparts.

We assessed whether individuals with higher educational attainment had perception of their weight status that were more ‘consistent’ with their actual weight than that of individuals with lower levels of education. Table 4 presents results from multinomial logit regression models for women and men with perceived weight as a dependent variable and actual weight status as one of the predictor variables. Education was a significant predictor of weight perception for women. Women with higher educational attainment were significantly less likely to perceive that they were slimmer than average and more likely to perceive that they were heavier than average controlling for their actual body weight status. There was no statistically significant difference in the relationship between education and weight perception across age groups (women in 20s and 30s versus others) among women.

**Table 4:** Coefficients from multinomial logit regression models for weight perception among women (*N* = 10,838) and men (N = 7109), 2008–2012

Characteristic	Women				Men			
	Model1^a^ Perceived Under weight	Perceived Over weight	Model2^a^ Perceived Under weight	Perceived Over weight	Model 1^a^Perceived Under weight	Perceived Over weight	Model2^a^ Perceived Under weight	Perceived Over weight
Educational attainment (middle school or less)^b^								
High school	−0.42**	0.45**	−0.49**	−0.02	−0.18	0.13	−0.03	0.10
College and above	−0.40**	0.38**	−0.44**	−0.17	−0.23	0.34*	0.11	0.41
BMI category (normal)								
Underweight	3.31**	−17.0	3.21**	−19.65	4.0**	1.52	3.06**	2.35
Overweight	−1.50**	2.87**	−1.59**	2.33**	−3.02**	2.84**	−2.42**	2.93**
Obesity	−0.58	4.91**	−0.72	4.14**	−2.07**	5.82 **	−2.06**	5.35**
Interaction								
High school*Underweight			0.23	0.62			15.65	0.44
High school*Overweight			0.21	0.55**			−1.08**	−0.04
High school*Obesity			−11.41	1.98**			0.05	0.55
College/above*Underweight			0.05	0.57			1.03	−14.51
College/above*Overweight			−0.21	0.94**			−0.73*	−0.15
College/above*Obesity			−11.7	1.65*			−0.15	0.88
Constant	−3.08**	0.11	−3.02**	0.60**	0.30	−2.2**	0.16	−2.22**

*p*-value ** < 0.01; * < 0.05. Age and survey year are controlled for in all models.

^a^People who considered themselves to be ‘average’ are a reference outcome.

^b^Omitted categories are shown in parentheses

**Fig. 1:**
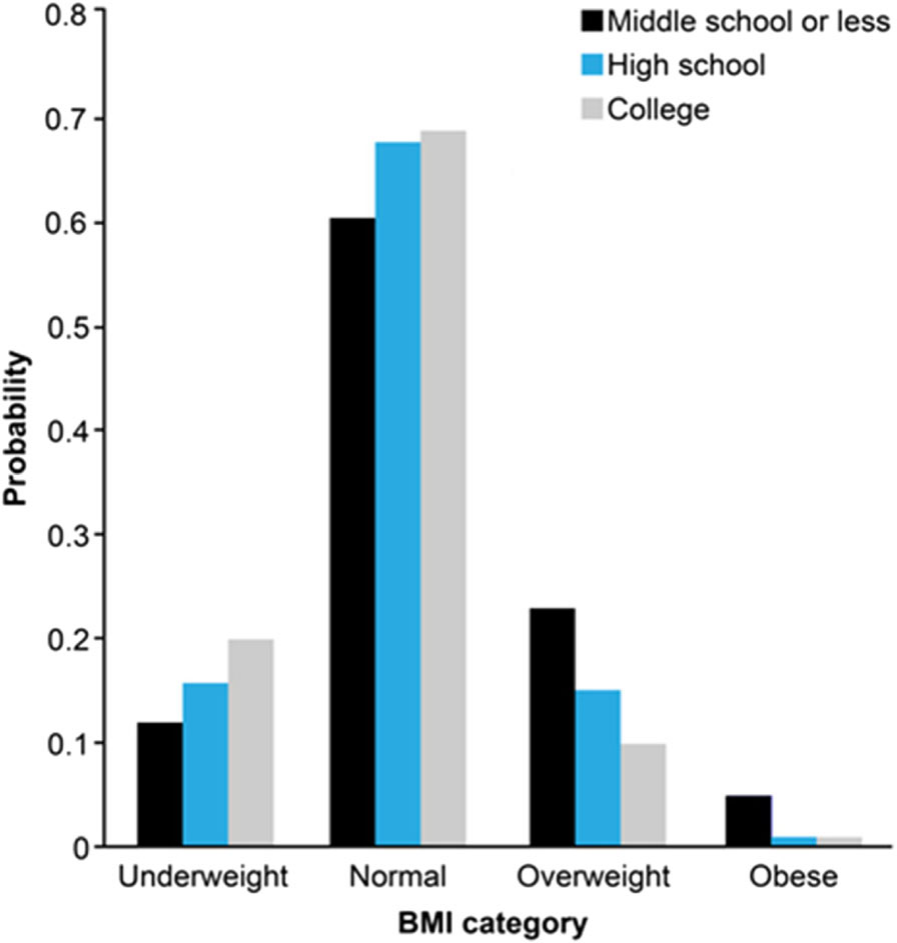
Predicted probability of weight perception of average among women. Notes: Weight perception refers to whether people perceive that they are of average weight. Model controls for age, survey year, education, weight status, and education*weight status

Figure 1 graphs predicted probabilities from the fully adjusted model with an interaction term between education and weight status for women who perceive that they are of average body size (Model 2 in Table 4). Most women in the normal weight category were likely to perceive that they were of average body size and the probability for the highest educated women was greater than that for the lowest educated. The pronounced educational differences were found among women who were overweight and under-estimated their body size. The probability for the women with college education who were overweight and considered their body size was normal was significantly lower than that for their counterparts with middle school level educations. The confidence intervals for the probability of under-perception of these two groups did not overlap, which means that the educational groups are significantly different.

**Fig. 2:**
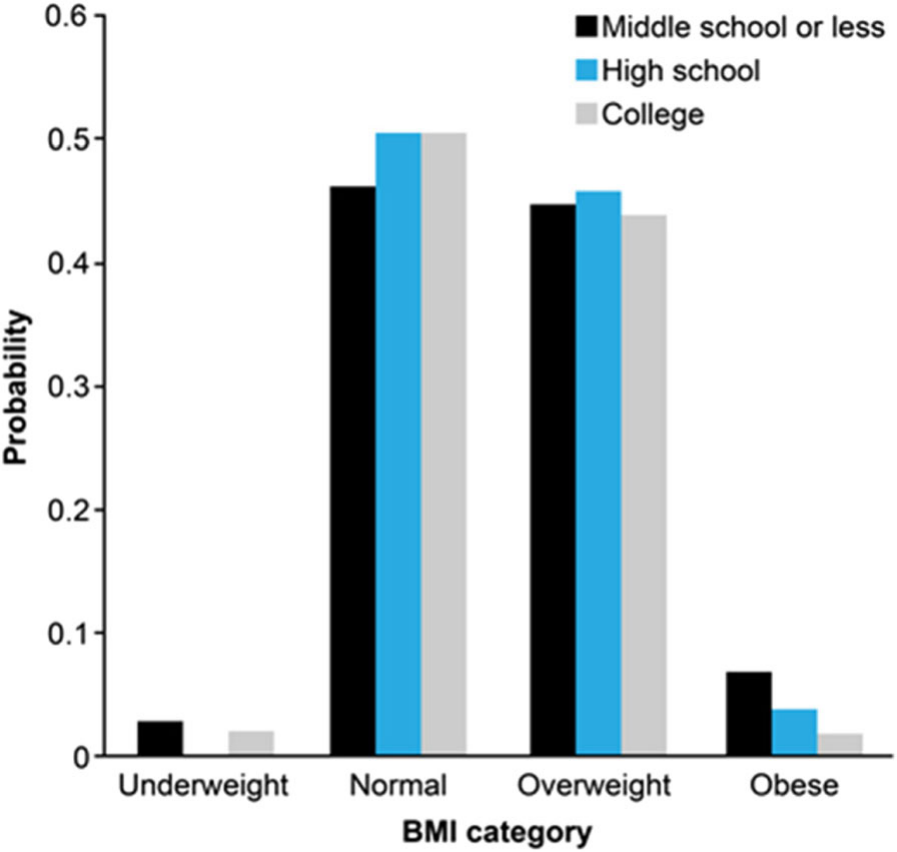
Predicted probability of weight perception of average among men. Notes: Weight perception refers to whether people perceive that they are of average weight. Model controls for age, survey year, education, weight status, and education*weight status

Table 4 and Fig. 2 show that compared to women, the educational gradients in weight perception were less pronounced for men. Figure 2 presents predicted probabilities for men who perceive that they are of average body size with different educational attainment across actual weight status categories. Figure 2 illustrates that men were more likely to under-perceive their body size than women. Weight perception was not strongly stratified among men across educational attainment, unlike women. In Fig. 2, around half of men who were overweight were likely to consider that they were of average body size regardless of their educational attainment.

## Discussion

Previous studies documented that the shape and strength of the relationship between education and weight are not always homogeneous across demographic groups within a country [2, 4, 5]. Extending previous findings, the current study demonstrates clear gender differences in the way education is related to body weight and weight perception. Korean men who have higher educational attainment are more likely to gain weight across the entire BMI distribution than men who have lower educational attainment but the relationship is reversed among women. Given that the positive relationship between education and obesity is not common in contemporary developed countries these clear gender differences in Korean society are interesting [6] Unlike women, highly educated men appeared to be heavier and the relationship was mostly explained by economic resources, particularly occupational status. Among men, behaviors among high-status workers are not consistent with a healthy lifestyle [34] and more highly educated men may thus not be socially motivated to engage in healthy behaviors [34, 46]. Korean culture encourages long working hours, e.g., the early bird catches the worm, and sedentary lifestyles are common in higher status occupations, e.g., high-ranking officials have private drivers etc. In addition, Korean men traditionally tend to place a high value on socializing, while viewing drinking positively as a crucial component to building masculine social networks [47]. Our finding that heavy drinking and long working hours tend to have a larger impact on men at the higher quantile of BMI supports that health behaviors play a crucial role in well-off men’s obesity status in particular.

Another possible explanation is that highly educated Korean women tend to be more sensitive to their body shape compared to men counterparts. Considering that Korean women have fewer opportunities for transferring educational attainment into economic resources than men due to the gender hierarchy, and women’s social status would be determined by their husband’s economic status, women may utilize their human capital to obtain symbolic resources such as physical attractiveness. Well-educated Korean women are more likely to accrue prestige by setting themselves apart with weight-related behaviors reflected by the boom in the fitness and plastic surgery industries in this country [48]. Education increases the demand for dieting although highly educated women have a lower objective “need,” but less educated women do not have the cultural means to afford special diets and are less likely to be motivated to override emerging obesogenic environments [49]. The finding shows that overweight women with lower educational attainment are likely to under-estimate their body size and this tendency is consistent across different age groups. These factors contribute to generating stronger educational inequalities in the ‘ideal slim look’ among Korean women compared to other developed countries. The social standards of gender roles may influence the ways men and women allocate their time to various activities during the day such that men with college educations are likely to spend more time at paid work, relative to women with college educations who are less likely to be in the labor market [50]. In a gendered society where the price of time use for non-paid work is much greater for men than women, men may have time constraints and spend less of their time engaged in leisure activities as a main breadwinner, particularly when they are working in higher-status jobs. In this condition, highly educated men are more likely to be exposed to obesity risk factors. Koreans also appear to have less aversion regarding large body size for men and tend to be less judgmental about men’s physical appearance [49]. Discrimination based on appearance in the labor and marriage market is relatively mild for men as compared to women. As we can see in Figs. 1 and 2, men were more likely to under-perceive their body size than women, that is, overweight men tend to consider themselves to be of ‘average’ weight, regardless of their educational attainment.

One of the main limitations of the study is the lack of longitudinal data on individuals’ body weight and body perception trajectories. Therefore, we cannot derive further inference about the causal effect of educational attainment on changes in weight status or weight control behaviors. It is also possible that the correlation between educational attainment and body weight and weight perceptions are derived from unobserved factors affecting both educational attainment and weight status. Future studies also need to consider gender differences in other important behavioral mediators, such as calorie intake, social capital, and psychological status, which were not included in current study due to the lack of reliable data.

## Conclusions

The current study examined the association between educational attainment and weight status, whether the association operated mainly through attained economic resources and health behaviors, and how these mechanisms varied by gender. Although education may help maintain a healthy weight in Western countries, it may not always be a protective factor in societies with different cultures. This research shed light on the intersection of gender and human capital that explains gender differences in health and health behaviors in context with structural gender-inequalities. It encourages future research on the nature of education as an important factor that helps people override unhealthy lifestyles in rapidly developing societies, especially those that have strong gender hierarchy.

## Appendix

**Table 5:** Coefficients from multinomial logit regression models for weight status among women, 2008–2012 (N = 10,838)

Characteristic	Model 1^a^ Under-weight	Over-weight	Obesity	Model 2^a^ Under-weight	Over-weight	Obesity	Model 3^a^ Under-weight	Over-weight	Obesity	Model 4^a^ Under-weight	Over-weight	Obesity
Educational attainment (middle school or less)^b^												
High school	0.70**	−0.74**	−0.80**	−0.15	−0.38**	−0.71**	−0.24	−0.39**	−0.69**	−0.22	−0.39**	−0.68**
	(0.21)	(0.06)	(0.09)	(0.24)	(0.08)	(0.11)	(0.25)	(0.08)	(0.12)	(0.24)	(0.08)	(0.12)
College and above	1.24**	−1.27**	−1.58**	0.01	−0.74**	−1.44**	−0.12	−0.73**	−1.42**	−0.12	−0.72**	−1.38**
	(0.20)	(0.07)	(0.11)	(0.25)	(0.09)	(0.15)	(0.27)	(0.10)	(0.15)	(0.27)	(0.10)	(0.15)
Household income (logged)							0.12	0.03	−0.16**	0.12	0.02	−0.16**
							(0.08)	(0.04)	(0.05)	(0.08)	(0.04)	(0.05)
Occupational status (manual worker)												
Professional							0.30	−0.08	0.24	0.26	−0.08	0.25
							(0.24)	(0.12)	(0.19)	(0.24)	(0.12)	(0.19)
Clerical/service/sales							0.39	0.06	0.25	0.39	0.06	0.25
							(0.22)	(0.09)	(0.14)	(0.22)	(0.09)	(0.13)
Not in the labor force							0.37	−0.02	0.15	0.32	0.00	0.18
							(0.21)	(0.08)	(0.12)	(0.21)	(0.08)	(0.12)
Marital status (married)												
Single				0.36*	−0.14	−0.35	0.38*	−0.14	−0.43*	0.42**	−0.17	−0.49*
				(0.15)	(0.11)	(0.19)	(0.16)	(0.11)	(0.19)	(0.16)	(0.12)	(0.20)
Separated/divorced/widowed				−0.12	−0.08	−0.06	−0.03	−0.07	−0.16	−0.01	−0.08	−0.22
				(0.26)	(0.09)	(0.14)	(0.27)	(0.10)	(0.14)	(0.27)	(0.10)	(0.14)
Health behaviors												
Hours of sleep										0.09	−0.07*	−0.16**
										(0.06)	(0.03)	(0.04)
Smoking (non-smoker)												
Current smoker										0.05	−0.20	−0.02
										(0.19)	(0.11)	(0.15)
Former smoker										−0.41	0.08	0.29
										(0.33)	(0.16)	(0.22)
Drinking (Never)												
Light drinker										0.03	0.01	0.04
										(0.14)	(0.07)	(0.10)
Light and frequent drinker										−0.15	−0.02	−0.01
										(0.16)	(0.08)	(0.12)
Moderate drinker										−0.49*	0.01	0.12
										(0.23)	(0.11)	(0.16)
Heavy drinker (60–80 g ethanol/a day)										0.04	0.33*	0.40
										(0.28)	(0.16)	(0.24)
Binge drinker (> 80 g ethanol/a day)										−0.02	0.51**	0.71**
										(0.32)	(0.20)	(0.25)
Constant	−3.00**	0.50**	−0.63**	0.41	−1.14**	−0.67*	−0.79*	−1.35**	0.44	−1.38	−0.88*	1.32*
	(0.18)	(0.05)	(0.07)	(0.49)	(0.21)	(0.31)	(0.79)	(0.35)	(0.50)	(0.90)	(0.43)	(0.60)

*p*-value ** < 0.01; * < 0.05. Age, survey year, self-reported health, region of residence, and parental education are controlled for in models 2–4. Robust standard errors are in parentheses.

^a^Normal weight is a reference outcome.

^b^Omitted categories are shown in parentheses

**Table 6:** Coefficients from multinomial logit regression models for weight status among men, 2008–2012 (N = 7109)

Characteristic	Model 1^a^ Under-weight	Over-weight	Obesity	Model 2^a^ Under-weight	Over-weight	Obesity	Model 3^a^ Under-weight	Over-weight	Obesity	Model 4^a^ Under-weight	Over-weight	Obesity
Educational attainment (middle school or less)^b^												
High school	−0.16	0.01	0.34**	−0.15	0.14	0.29*	−0.03	0.06	0.20	−0.05	0.05	0.18
	(0.24)	(0.09)	(0.13)	(0.26)	(0.10)	(0.14)	(0.27)	(0.10)	(0.15)	(0.27)	(0.10)	(0.15)
College and above	−0.22	0.12	0.42**	−0.22	0.28**	0.34*	0.03	0.08	0.13	0.02	0.08	0.11
	(0.24)	(0.08)	(0.13)	(0.29)	(0.10)	(0.16)	(0.30)	(0.11)	(0.17)	(0.30)	(0.11)	(0.18)
Household income (logged)							−0.14	0.12**	0.10	−0.11	0.12**	0.10
							(0.07)	(0.04)	(0.06)	(0.07)	(0.04)	(0.06)
Occupational status (manual worker)												
Professional							−0.30	0.31**	0.32*	−0.26	0.29**	0.26
							(0.30)	(0.10)	(0.14)	(0.30)	(0.10)	(0.14)
Clerical/service/sales							−0.48	0.21*	0.25*	−0.46	0.19*	0.20
							(0.27)	(0.09)	(0.12)	(0.27)	(0.09)	(0.13)
Not in the labor force							0.22	0.05	−0.22	0.18	0.10	−0.12
							(0.27)	(0.11)	(0.17)	(0.27)	(0.11)	(0.17)
Marital status (married)												
Single				0.16	−0.35**	−0.34*	−0.08	−0.26*	−0.19	−0.02	−0.25	−0.18
				(0.29)	(0.10)	(0.14)	(0.31)	(0.11)	(0.14)	(0.31)	(0.11)	(0.15)
Separated/divorced/widowed				0.41	0.01	−0.03	0.18	0.12	0.12	0.25	0.08	−0.02
				(0.41)	(0.16)	(0.24)	(0.44)	(0.16)	(0.25)	(0.45)	(0.16)	(0.25)
Health behaviors												
Hours of sleep										0.02	−0.09*	−0.25**
										(0.10)	(0.03)	(0.05)
Smoking (current smoker)												
Non smoker										−0.62*	0.03	0.07
										(0.29)	(0.09)	(0.13)
Former smoker										−0.08	0.25**	0.09
										(0.31)	(0.09)	(0.14)
Drinking (Never)												
Light drinker										0.22	−0.10	−0.16
										(0.33)	(0.12)	(0.18)
Light and frequent drinker										0.06	−0.16	−0.55**
										(0.33)	(0.12)	(0.19)
Moderate drinker										−0.19	0.19	0.01
										(0.34)	(0.12)	(0.18)
Heavy drinker (60–80 g ethanol/a day)										−0.49	0.12	0.00
										(0.37)	(0.12)	(0.18)
Binge drinker (> 80 g ethanol/a day)										−0.66	0.42**	0.71**
										(0.38)	(0.12)	(0.17)
Constant	−2.44**	0.41**	−1.16**	−0.99	−0.05	−0.14	0.24	−1.08**	−1.03	0.30	−0.59	0.44
	(0.19)	(0.07)	(0.11)	(0.72)	(0.25)	(0.36)	(0.96)	(0.40)	(0.59)	(1.23)	(0.48)	(0.72)

*p*-value ** < 0.01; * < 0.05. Age, survey year, self-reported health, region of residence, and parental education are controlled for in models 2–4. Robust standard errors are in parentheses.

^a^Normal weight is a reference outcome.

^b^Omitted categories are shown in parentheses

## Abbreviations

BMI: Body Mass Index
KNHANES: Korean National Health and Nutrition Examination Survey
OECD: Organisation for Economic Co-operation and Development
OR: Odds Ratio
WHO: World Health Organization
